# Screening of a 4-Ethylguaiacol-Producing *Bacillus coagulans* JN11 and Biochemical Characterization of Its Phenolic Acid Decarboxylase BcPAD

**DOI:** 10.3390/microorganisms14061338

**Published:** 2026-06-15

**Authors:** Yufeng Liu, Hao Wang, Xinyue Lan, Rui Wang, Can Liu, Jun Liu, He Zou, Siqi Yuan

**Affiliations:** 1 School of Food and Liquor Engineering, Sichuan University of Science & Engineering, Yibin 644000, China; liuyufeng6220245@163.com (Y.L.); kingh212@163.com (H.W.); lanxinyue135400@163.com (X.L.); wangrui0693@163.com (R.W.); liucan13350108317@163.com (C.L.); liujunbio@suse.edu.cn (J.L.); 18381307626@163.com (H.Z.); 2 Brewing Science and Technology Key Laboratory of Sichuan Province, Sichuan University of Science & Engineering, Yibin 644000, China

**Keywords:** 4-ethylguaiacol, *Bacillus coagulans*, phenolic acid decarboxylase, enzymatic characterization, substrate specificity, molecular docking

## Abstract

4-Ethylguaiacol (4-EG) is a volatile phenolic compound associated with smoky, woody, and spicy aroma notes in fermented foods and beverages, including Baijiu. In this study, a 4-EG-producing strain, designated JN11, was obtained by screening isolates from Baijiu pit mud and identified as *Bacillus coagulans* based on morphological, physiological, biochemical, and 16S rRNA analyses. In sorghum juice medium, strain JN11 produced 271.6 ± 2.7 μg/L 4-EG. To investigate the upstream decarboxylation step involved in volatile phenol formation, the phenolic acid decarboxylase gene, *BcPAD*, was cloned and heterologously expressed in *Escherichia coli* BL21(DE3). The *BcPAD* gene comprises 504 bp and encodes a 167-amino-acid protein. Recombinant BcPAD exhibited maximal activity at pH 6.0 and 50 °C and retained more than 60% residual activity after 5 h at 30–40 °C. Fe^3+^ increased enzyme activity to 115.36% of the control, whereas Zn^2+^ markedly inhibited enzyme activity and SDS completely inactivated the enzyme. BcPAD showed the highest activity toward *p*-coumaric acid, with a specific activity of 460.6 ± 18.3 U/mg and a catalytic efficiency (*Kcat*/*Km*) of 12.1 ± 1.4 mM^−1^·s^−1^, while lower activities were observed toward caffeic acid and ferulic acid, and no activity was detected toward sinapic acid. Homology modeling and molecular docking suggested that the superior catalytic performance toward *p*-coumaric acid may be related to favorable hydrogen-bonding interactions and substrate orientation within the active site. Although 4-EG production was observed during fermentation by strain JN11, BcPAD was biochemically characterized as a phenolic acid decarboxylase likely responsible for the upstream formation of vinyl derivatives in the proposed pathway. These findings improve our understanding of phenolic acid decarboxylases from *B. coagulans* and provide a basis for further investigation of the roles of strain JN11 and BcPAD in volatile phenol formation during Baijiu production.

## 1. Introduction

4-Ethylguaiacol (4-EG) is an important volatile phenolic flavor compound with characteristic smoky, woody, spicy, and slightly sweet notes [1] and is widely found in fermented foods such as Baijiu, soy sauce, and pickles [2]. In Chinese Baijiu, 4-EG is considered one of the key trace aroma-active compounds contributing to the Jiang-flavor style, and at appropriate concentrations, it enhances the mellow mouthfeel of the liquor [3]. In addition to its sensory contribution, 4-EG also exhibits notable reactive oxygen species scavenging capacity and anti-inflammatory effects, showing potential physiological activities in aspects such as antioxidation, cardiovascular disease prevention, and the treatment of central nervous system disorders [4,5,6]. Therefore, 4-EG is not only an important flavor compound in Baijiu but also a potential health-related factor and has been widely applied in industries such as alcoholic beverages, soy sauce, and tobacco [3,7]. Currently, the main approaches for 4-EG production include natural extraction, chemical synthesis, and biotransformation [8]. Compared with natural extraction, which is limited by low extraction efficiency and restricted raw material availability, and chemical synthesis, which involves harsh reaction conditions and greater environmental burdens, biotransformation has attracted widespread attention because of its mild conditions, high selectivity, and environmental friendliness [9].

**Figure 1:**
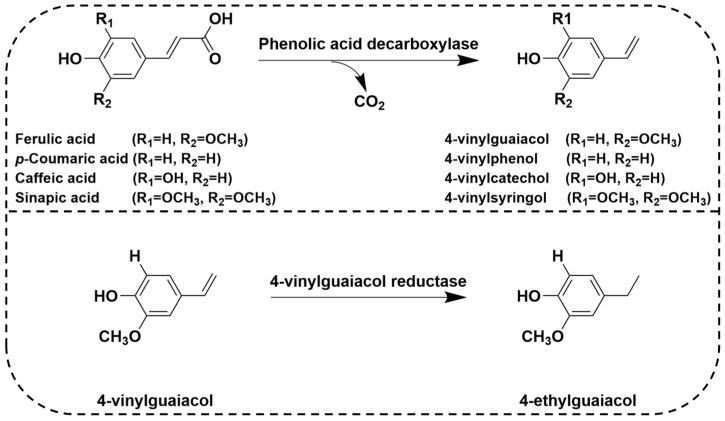
Biosynthetic pathway for the formation of 4-vinyl derivatives and 4-ethylguaiacol from hydroxycinnamic acids.

Phenolic acids mainly include hydroxycinnamic acid compounds such as ferulic acid, *p*-coumaric acid, caffeic acid, and sinapic acid [10]. These compounds are widely present in plant cell walls and agro-industrial by-products, usually in free or bound forms, and can be released by the action of esterases or other lignocellulose-degrading enzymes [11]. Phenolic acid decarboxylase (PAD, EC 4.1.1.102) catalyzes the non-oxidative decarboxylation of these hydroxycinnamic acids to produce 4-vinyl derivatives [12,13,14], including 4-vinylguaiacol (4-VG), which are generally of high economic value [15,16]. Among these products, 4-VG can be further converted into 4-EG by reductases (Figure 1) [10]. Therefore, as a key enzyme in the biosynthetic pathway of 4-EG, PAD-based biotransformation strategies show promising prospects for the sustainable production of natural flavor compounds and the valorization of agro-industrial residues. Previous studies have shown that PAD is mainly found in bacteria, fungi, and a few plants [17]. PADs from different sources exhibit significant differences in enzymatic and biochemical properties. For example, the optimal pH of dLPPAD from *Pichia pastoris* is 7.0 [12], whereas the optimal pH of FADase from *Enterobacter* sp. Px6-4 is 4.0 [18]. In addition, BsPAD from *Bacillus subtilis* exhibits an optimal reaction temperature of 50 °C [19], whereas CgPAD from *Candida guilliermondii* exhibits maximal activity at 25 °C [20]. Overall, bacterial PADs generally display high catalytic activity, while PADs from *Bacillus* species possess advantages in thermal stability and environmental tolerance [17], highlighting their considerable potential in flavor biomanufacturing and enzyme engineering. In recent years, related studies have gradually expanded from strain screening to gene cloning, heterologous expression, enzymatic characterization, and catalytic mechanism analysis [12,21,22,23,24,25]. However, current research has mainly focused on lactic acid bacteria, yeasts, and a few model strains, and systematic studies on 4-EG-producing functional microorganisms and their key enzyme, PAD, in the Baijiu fermentation system remain limited. In particular, in-depth studies on the molecular characteristics, enzymatic properties, and substrate recognition mechanism of PAD derived from *B. coagulans* are still lacking.

*Daqu* (fermentation starter) and pit mud are complex microbial ecosystems enriched in functional microorganisms closely associated with flavor formation [26]. Among these microorganisms, members of the genus *Bacillus* are regarded as important aroma-producing microbes during Baijiu fermentation due to their high heat resistance, strong environmental adaptability, and considerable metabolic diversity [27]. However, despite increasing interest in volatile phenol biosynthesis, systematic studies on 4-EG-producing functional microorganisms and their key phenolic acid decarboxylases in the Baijiu fermentation system remain limited, particularly with respect to *B. coagulans*. Therefore, in the present study, *Bacillus* strains were isolated and screened from *Daqu* and pit mud, and a 4-EG-producing strain, designated JN11, was identified as *B. coagulans*. To investigate the upstream decarboxylation step potentially associated with volatile phenol formation, BcPAD was cloned, heterologously expressed, and biochemically characterized. Homology modeling and molecular docking were further conducted to explore structural factors that may underlie its substrate preference. This study improves our understanding of BcPAD as a phenolic acid decarboxylase from *B. coagulans* and provides a basis for further investigation of the roles of strain JN11 and BcPAD in volatile phenol formation during Baijiu production.

## 2. Materials and Methods

### 2.1. Strains, Plasmids and Chemicals

Information on the nine bacterial strains and three plasmids used in this study is presented in Table 1. The bacterial genomic DNA extraction kit was purchased from Labgic Technology Co., Ltd. (Beijing, China). DNA polymerase, T4 DNA ligase, BamH I and Hind III restriction endonucleases, and DNA and protein molecular weight markers were purchased from TransGen Biotech Co., Ltd. (Beijing, China). Kanamycin (Kan) and isopropyl-β-D-thiogalactopyranoside (IPTG) were purchased from Solarbio Science & Technology Co., Ltd. (Beijing, China). pentyl acetate, ethylene diamine tetraacetic acid (EDTA), sodium dodecyl sulfate (SDS), 4-ethylguaiacol, ferulic acid (FA), *p*-coumaric acid (PCA), caffeic acid (CA), and sinapic acid (SA) were purchased from Yuanye Biotechnology Co., Ltd. (Shanghai, China).

### 2.2. Screening of 4-EG-Producing Strains

A total of 100 *Bacillus* spp. strains preserved in our laboratory were used for screening, including 37 strains from high-temperature *Daqu*, 41 strains from medium-temperature *Daqu*, and 22 strains from liquor cellar. All strains were individually streaked onto de Man, Rogosa and Sharpe (MRS) agar plates and incubated at 37 °C for 16 h to reactivate the cultures. Single colonies were then selected and inoculated into LB broth and cultured at 37 °C with shaking at 180 rpm for 16 h. For primary screening, the seed cultures at an inoculum size of 1 × 10^6^ CFU/mL were inoculated into the sorghum juice fermentation medium [28,29]. After 7 days of fermentation, the volatile compounds and 4-EG concentration in the fermentation products were analyzed using gas chromatography-mass spectrometry (GC-MS). Strains showing detectable 4-EG production were considered positive candidates. Among the 100 strains screened, five strains, designated JXQ2, JXQ5, NXQ4, JN2, and JN11, were identified as 4-EG-positive strains and were subjected to secondary screening. These five strains were further compared for their 4-EG production under identical fermentation conditions, and the strain with the highest 4-EG yield was selected for subsequent experiments.

The fermentation broth was centrifuged at 8000 rpm for 5 min, and the resulting supernatant was filtered through a 0.22 μm membrane filter. Then, 1 mL of the filtrate was transferred into a 15 mL headspace bottle, and 20 μL of the internal standard solution (10 g/L pentyl acetate) was added. The sample was heated in a 60 °C metal bath for 15 min, after which the extraction fiber was inserted into the headspace bottle for adsorption extraction for 45 min. After extraction, the volatile compounds were analyzed by GC-MS. GC conditions were as follows: a DB-WAX capillary column (30 m × 0.25 mm, 0.25 µm) was used for manual injection, and the injector temperature was maintained at 230 °C. The oven temperature program was as follows: the initial temperature was set at 65 °C and held for 3 min, then increased to 150 °C at a rate of 5 °C/min and held for 2 min, followed by a further increase to 230 °C at 10 °C/min, where it was maintained for 10 min. Helium was used as the carrier gas at a flow rate of 0.8 mL/min, with a split ratio of 20:1 and an injection volume of 1 μL. MS conditions were as follows: electron ionization was employed, the mass scan range was *m*/*z* 20–500, the electron energy was 70 eV, the ion source temperature was 230 °C, the quadrupole temperature was 150 °C, and the solvent delay time was 3 min.

### 2.3. Identification and Physiological Characterization of the 4-EG-Producing Strain

Strain identification was based on colony morphology, biochemical characteristics, and molecular phylogenetic analysis. Morphological observation and biochemical characterization of the strain were conducted according to Bergey’s Manual of Determinative Bacteriology [30]. Molecular phylogenetic analysis was performed according to a previously reported method [31]. Genomic DNA was extracted from the strain using a bacterial genomic DNA extraction kit, and the 16S rRNA gene was amplified using the primers 27F (5′-AGAGTTTGATCCTGGCTCAG-3′) and 1492R (5′-GGTTACCTTGTTACGACTT-3′). The PCR products were examined by 1% (*w*/*v*) agarose gel electrophoresis and then sent to Youkang Biotechnology Co., Ltd. (Chengdu, China) for sequencing. The obtained sequences were subjected to BLAST (https://blast.ncbi.nlm.nih.gov/Blast.cgi; accessed on 10 January 2026) homology analysis, and a phylogenetic tree was constructed using the neighbor-joining method in MEGA 6.0 software with 1000 random bootstrap replications.

To determine the growth curve of the strain, the activated culture was inoculated into MRS broth at 1% (*v*/*v*) and incubated at 37 °C with shaking at 180 rpm. The optical density (OD) 600 of the culture was measured hourly for 36 h, with uninoculated MRS broth used as the blank control. To evaluate strain tolerance under different environmental conditions, the activated culture was inoculated into MRS broth at 1% (*v*/*v*) and incubated with shaking at 180 rpm for 12 h under different pH values (4, 5, 6, 7, 8, and 9), temperatures (20, 25, 30, 35, 40, and 45 °C), ethanol concentrations (0, 2, 4, 6, 8, and 10%, *v*/*v*), sodium chloride concentrations (0, 2, 4, 6, 8, and 10%, *w*/*v*), and glucose concentrations (0, 5, 10, 15, 20, and 25%, *w*/*v*). The OD600 was then measured. All experiments were performed in triplicate.

### 2.4. Cloning and Sequence Analysis of the BcPAD Gene

Based on the sequence information for the phenolic acid decarboxylase gene of *Bacillus coagulans* available in the NCBI database (GenBank accession number: WP_263931213.1), full-length amplification primers were designed: BcPAD-BamHI-F (5′-CGGGATCCATGAAAACATTAGAAGAA-3′) and BcPAD-HindIII-R (5′-CCCAAGCTTTTATGAAAATTTTAGTTTGAA-3′). PCR amplification was performed using the genomic DNA of *Bacillus coagulans* JN11 as the template under the following conditions: pre-denaturation at 95 °C for 4 min; 34 cycles of denaturation at 95 °C for 40 s, annealing at 55 °C for 40 s, and extension at 72 °C for 1 min; followed by a final extension at 72 °C for 10 min. The amplified product was verified by 1% (*w*/*v*) agarose gel electrophoresis and then purified. The purified target fragment and the pET-28a vector were double-digested with BamHI and HindIII, respectively. After purification of the digested products, ligation was performed using T4 DNA ligase at 25 °C for 4 h. The ligation product was transformed into *E. coli* BL21(DE3) cells, and positive colonies identified by colony PCR were sent to Youkang Biotechnology Co., Ltd. (Chengdu, China) for sequencing. Following BLAST analysis of the sequencing results, the amino acid sequence was deduced using DNAMAN 6.0 software. The basic physicochemical properties of the deduced amino acid sequence were analyzed using the ProtParam tool (https://web.expasy.org/protparam/, accessed on 12 January 2026), and multiple sequence alignment was performed using Clustal 1.81 software and ESPript 3.2 (https://espript.ibcp.fr/ESPript/cgi-bin/ESPript.cgi, accessed on 15 January 2026).

### 2.5. Heterologous Expression and Purification of Recombinant BcPAD

A single colony of the recombinant *E. coli* BL21(DE3)/pET-28a-*BcPAD* strain was picked and inoculated into Luria–Bertani (LB) medium containing 50 µg/mL kanamycin, followed by cultivation at 37 °C and 180 rpm for 12 h. The seed culture was then inoculated at 1% (*v*/*v*) into 100 mL of LB medium containing 50 µg/mL kanamycin and incubated at 37 °C and 180 rpm until the OD600 reached 0.6–0.8. IPTG was added to final concentrations of 0.0, 0.1, 0.3, 0.5, 0.7, and 1.0 mmol/L, and protein expression was induced for 3 h at 180 rpm to determine the optimal IPTG concentration. Cells were collected by centrifugation at 8000 rpm for 5 min, and protein expression levels were evaluated by sodium dodecyl sulfate-polyacrylamide gel electrophoresis (SDS-PAGE). In addition, under the optimal IPTG concentration, protein expression was induced for 16 h at different temperatures (25, 30, and 37 °C) at 180 rpm to identify the optimal temperature for soluble protein expression. Cells were harvested by centrifugation at 8000 rpm for 5 min at 4 °C and resuspended in 10 mL of 50 mmol/L sodium phosphate buffer (Na_2_HPO_4_–NaH_2_PO_4_, pH 7.0). The cells were disrupted by ultrasonication (180 W; 3 s on, 3 s off) for 15 min in an ice bath. The supernatant was collected by centrifugation at 10,000 rpm for 5 min at 4 °C. The sediment was treated with 8 mol/L urea at 4 °C for 30 min. The distribution of the target protein in the supernatant and sediment was analyzed by SDS-PAGE. The supernatant was further purified by Ni-NTA affinity chromatography, and the target protein was eluted with 100 mmol/L imidazole. The purified protein was analyzed by SDS-PAGE and subsequently used for enzyme activity assays. The concentration of the purified protein was determined using the Bradford method [32].

### 2.6. Determination of Recombinant BcPAD Enzyme Activity

The standard reaction mixture consisted of 0.8 mL of Na_2_HPO_4_–citrate buffer (pH 6.0), 0.1 mL of 50 mmol/L FA, and 0.1 mL of 0.1354 mg/mL purified BcPAD. After incubation in a 37 °C water bath for 5 min, the reaction was terminated by the addition of 2 mL of methanol. The resulting mixture was filtered through a 0.22 μm membrane filter, and the concentration of 4-vinyl derivatives was determined by high-performance liquid chromatography (HPLC). The control was prepared by replacing the enzyme solution with 0.1 mL of Na_2_HPO_4_–citrate buffer. The HPLC conditions were as follows: a PotenSil-C18 column (4.6 mm × 150 mm, 5 μm, Anjie, Chengdu, China), an injection volume of 5 μL, a flow rate of 1.0 mL/min, a column temperature of 30 °C, a mobile phase of methanol/0.1% acetic acid (40:60, *v*/*v*), and a detection wavelength of 260 nm. One unit of enzyme activity (U) was defined as the amount of enzyme required to produce 1 μmol of 4-vinyl derivative per minute.

### 2.7. Effects of pH, Temperature, Metal Ions, and Chemical Reagents on the Activity of BcPAD

The optimal pH of the enzyme was determined over a pH range of 5.0–8.0 using Na_2_HPO_4_–citrate buffer under the standard assay conditions described above. At the optimal pH, the optimal temperature of the enzyme was determined over a range of 20–70 °C. The maximum enzyme activity was defined as 100%, and the relative activity under each condition was calculated accordingly.

pH stability was evaluated by measuring residual activity after incubating the enzyme without substrate in Na_2_HPO_4_–citrate buffer at pH 5.0–8.0 at room temperature for 0–5 h. Thermal stability was evaluated by measuring the residual activity after incubating the enzyme in Na_2_HPO_4_–citrate buffer (pH 6.0) at 20–70 °C for 0–5 h. Under the same pH or temperature conditions, the activity of the non-incubated enzyme was defined as 100%, and the relative activity at different incubation times was calculated accordingly.

Under optimal assay conditions, various metal ions (Ba^2+^, Ca^2+^, Co^2+^, Cs^+^, Cu^2+^, Fe^3+^, Li^+^, Mg^2+^, Mn^2+^, Ni^2+^, and Zn^2+^) and chemical reagents (EDTA, SDS, and urea) were individually added to the reaction mixture at a final concentration of 1 mmol/L, whereas Triton X-100 (Yuanye Biotechnology Co., Ltd., Shanghai, China) and ethanol were added at a final volume fraction of 1% to evaluate their effects on the enzyme activity. The reaction mixture without added metal ions or chemical reagents served as the control, and its enzyme activity was defined as 100%, relative to which all other activities were calculated.

### 2.8. Determination of the Substrate Specificity and Kinetic Parameters of BcPAD

Substrate specificity of BcPAD was evaluated using FA, PCA, CA, or SA at a final concentration of 80 mM under the optimal reaction conditions. Enzyme activity toward each substrate was expressed as specific activity (U/mg protein).

For kinetic analysis, initial reaction rates were measured under the optimal reaction conditions for 5 min in Na_2_HPO_4_–citrate buffer containing varying concentrations (5–160 mM) of FA, PCA, or CA. Nonlinear regression was performed by fitting the data to the Michaelis–Menten equation using GraphPad Prism 10.1 software, and the Michaelis constant (*Km*), apparent maximum velocity (*Vmax*), turnover number (*Kcat*), and *Kcat*/*Km* values for BcPAD with each substrate were calculated, respectively.

### 2.9. Homology Modeling and Molecular Docking of BcPAD

The crystal structure of FADase from *Lactobacillus plantarum* (PDB ID: 2W2A) was selected as the template for homology modeling of BcPAD in SWISS-MODEL (https://swissmodel.expasy.org/) based on amino acid sequence alignment. The structural information for the ligands FA, PCA, and CA was obtained from the PubChem database (https://pubchem.ncbi.nlm.nih.gov/). AutoDock Tools was used to remove water molecules and metal ions and to add hydrogen atoms. Molecular docking was performed using AutoDock Vina 1.2.7 software, with the grid box dimensions set to 39 × 36 × 36 and the center coordinates set to x = 2.252, y = 30.032, and z = 25.138. The docking results were evaluated based on binding energy and interaction patterns, and the conformation with the lowest binding energy was selected as the final docking result. PyMOL 2.5 software was used for three-dimensional visualization of the tertiary structure and molecular docking results, and Discovery Studio 2025 software was used for two-dimensional visualization of protein–ligand interactions.

### 2.10. Statistical Analysis

Data are presented as the mean ± standard error (SE) from three independent experiments. Differences among samples were analyzed using one-way analysis of variance (ANOVA) followed by Duncan’s multiple range test in SPSS 27 software. Differences were considered significant at *p* < 0.05. Data were visualized using Origin 2024 software.

## 3. Results and Discussion

### 3.1. Screening and Analysis of 4-EG-Producing Strains

**Figure 2:**
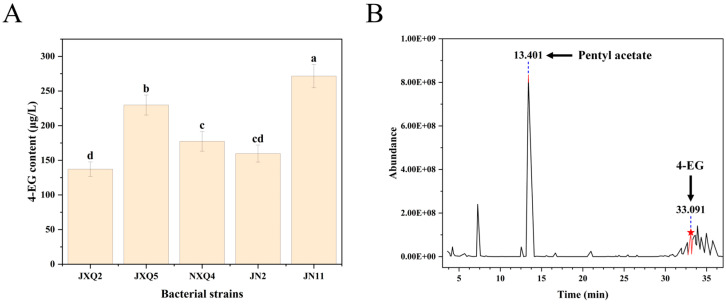
Analysis of 4-EG production by strains. (**A**) 4-EG production by different strains after 7 days of fermentation. (**B**) GC-MS chromatogram of the JN11 strain after 7 days of fermentation. Data are presented as the mean ± SE of three independent experiments. Different letters indicate significant differences according to Duncan’s multiple range test at *p* < 0.05. The red star indicates the peak corresponding to 4-EG and is used for visual emphasis only.

Using 4-EG yield as the screening indicator, GC-MS analysis showed that five of the 100 *Bacillus* strains (JXQ2, JXQ5, NXQ4, JN2, and JN11) produced 4-EG during fermentation in sorghum juice medium (Figure 2A). The fermentation product of the JN11 strain exhibited a retention time of 33.091 min for 4-EG (Figure 2B, Table S1), achieving the highest yield among all strains at 271.6 ± 2.7 μg/L. This yield surpasses the 245.3 μg/L obtained by *Bacillus cereus* wsp-2-2 utilizing wheat bran as a substrate [3] but is lower than the 460.0 μg/L obtained from *Saccharomyces fibuligera* B0 using malt juice as a fermentation medium [33]. The observed difference in yield may be attributed to the varying synthesis capabilities of the strains for 4-EG, as well as the differences in the fermentation media employed. Notably, it has been confirmed that specific strains of *Bacillus licheniformis* [34] and *Bacillus subtilis* [35] lack the capability to synthesize 4-EG. Based on the 4-EG production capabilities of the aforementioned strains, the JN11 strain was ultimately chosen for further identification, biological characterization, and functional studies on phenolic acid decarboxylase.

### 3.2. Identification of JN11 Strain

**Figure 3:**
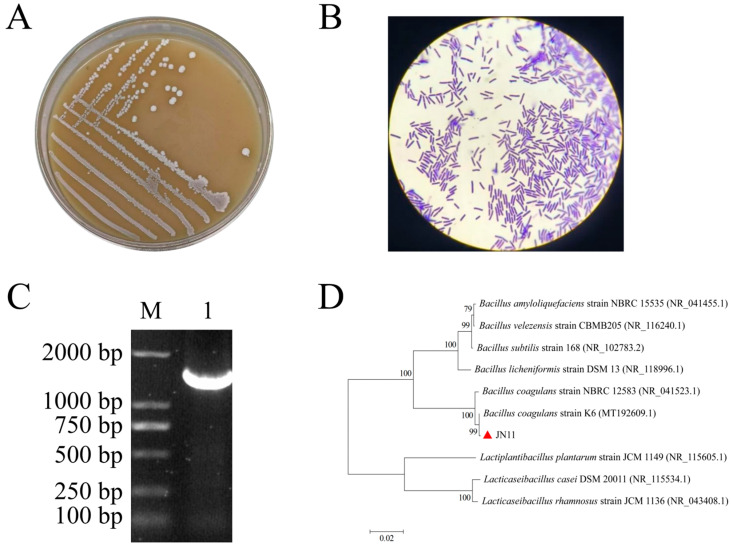
The morphological and genetic characteristics of the JN11 strain. (**A**) The morphology of the JN11 strain on MRS medium. (**B**) The Gram staining result of the JN11 strain (1000×). (**C**) PCR amplification product of the 16S rRNA gene from the JN11 strain. (**D**) The phylogenetic tree of the JN11 strain. The red triangle indicates the target strain.

The JN11 strain was inoculated onto MRS medium to examine the morphology of a single colony (Figure 3A). It was observed that the colony exhibited a circular shape, a moist surface, slight elevation, and an opaque milky-white color. Following Gram staining, microscopic examination revealed that the JN11 strain appeared blue-violet, indicating that it is a Gram-positive bacterium with a relatively short rod-shaped morphology and rounded ends (Figure 3B). The biochemical characteristics of the JN11 strain are detailed in Table 2. The JN11 strain can ferment glucose, sucrose, and lactose, except for mannitol. Additionally, it exhibits activities of catalase, cellulase, and protease. The strain was positive in gelatin and starch hydrolysis, nitrate reduction, VP test, and anaerobic growth test but negative in citrate utilization and indole test. The 16S rRNA gene of the JN11 strain was amplified by PCR (Figure 3C) and then sequenced, and the results indicated that the sequence similarity between the JN11 strain and *B. coagulans* reached 99.44%. The phylogenetic analysis further indicated that the genetic distance between the JN11 strain and *B. coagulans* K6 was the closest (Figure 3D), so it was designated as *B. coagulans* JN11. The 16S rRNA gene sequence of strain JN11 has been deposited in the NCBI GenBank database under accession number PZ463283.1.

### 3.3. Physiological Characteristics of Bacillus coagulans JN11

**Figure 4:**
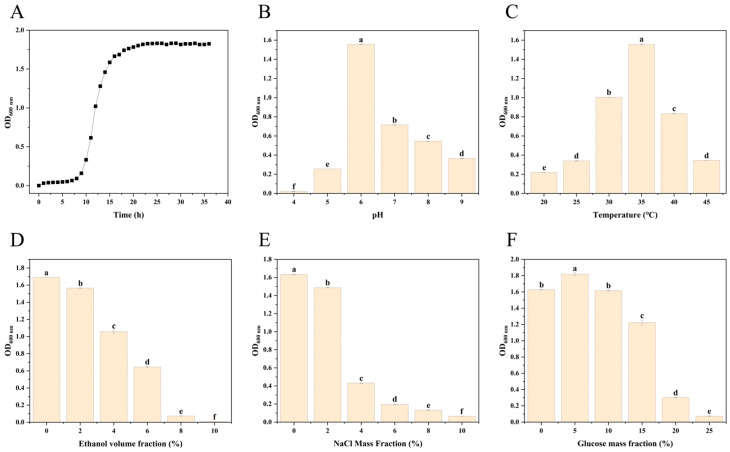
Growth characteristics of *B. coagulans* JN11 under different conditions. (**A**) Growth curve of *B. coagulans* JN11. (**B**) Effect of pH on the growth of *B. coagulans* JN11. (**C**) Effect of temperature on the growth of *B. coagulans* JN11. (**D**) Effect of ethanol on the growth of *B. coagulans* JN11. (**E**) Effect of NaCl on the growth of *B. coagulans* JN11. (**F**) Effect of glucose on the growth of *B. coagulans* JN11. Data are presented as the mean ± SE of three independent experiments. Different letters indicate significant differences according to Duncan’s multiple range test at *p* < 0.05.

Growth characterization experiments indicated that *B*. *coagulans* JN11 remained in the lag phase from 0 to 7 h, entered the logarithmic phase between 8 and 15 h, transitioned into the stationary phase from 16 to 25 h, and subsequently entered the decline phase after 25 h (Figure 4A). The strain grew over a pH range of 5 to 9, with an optimal pH of 6.0 (Figure 4B). *B*. *coagulans* JN11 grew over a temperature range of 20–45 °C, with the optimal growth temperature being 35 °C (Figure 4C). At ethanol volume fractions of 2–10%, growth was significantly inhibited, and when the ethanol volume fraction exceeded 8%, the strain almost ceased to grow (Figure 4D). When the NaCl concentration reached 4%, the OD of *B*. *coagulans* JN11 was only 26.44% of that of the control (Figure 4E). Notably, the addition of 5% glucose further stimulated growth, increasing the OD by 11.75% (Figure 4F). However, when the glucose concentration exceeded 15%, the growth of strain JN11 was significantly inhibited, although its relative OD still remained at 75.19%. In summary, *B*. *coagulans* JN11 prefers a mildly acidic, low-sugar environment and exhibits a certain tolerance to high temperature and ethanol, but is intolerant of high-salt conditions.

### 3.4. Gene Cloning and Bioinformatics Analysis of BcPAD

**Figure 5:**
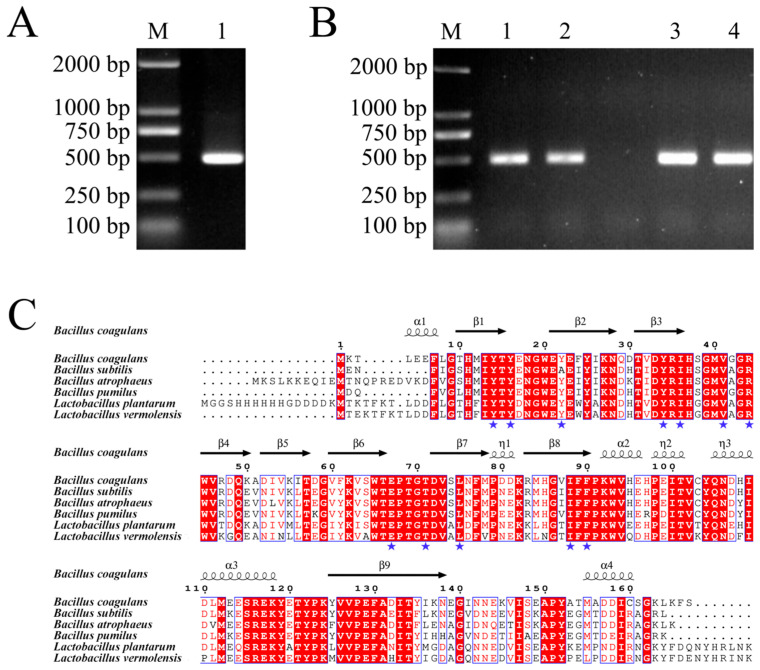
Gene cloning and multiple sequence alignment analysis of BcPAD. (**A**) PCR amplification of the *BcPAD* gene. M: DNA DL2000 Marker; Lane 1: PCR amplification product. (**B**) PCR identification of colonies from *E. coli* BL21(DE3)-pET28a-*BcPAD*. M: DNA DL2000 Marker; Lanes 1–4: Colony PCR products. (**C**) Sequence alignment of BcPAD with other identified phenolic acid decarboxylase. Consistent amino acid sequences are highlighted with a red background; active sites are marked with blue pentagrams.

Amplification was conducted using DNA from *B*. *coagulans* JN11 as a template, with primers BcPAD-BamHI-F and BcPAD-HindIII-R, resulting in the detection of a band approximately 500 bp in size through agarose gel electrophoresis (Figure 5A). Sequencing and comparative analyses confirmed that the full-length sequence of the *BcPAD* gene is 504 bp, comprising a complete open reading frame encoding 167 amino acids (Figure S1), which is similar in size to the 166 amino acids encoded by BlPAD from *Bacillus licheniformis* [36]. PCR identification was performed on transformed *E. coli* BL21(DE3) colonies harboring the pET28a-*BcPAD* ligation product. The results revealed four bands that corresponded to the expected size (Figure 5B), indicating the presence of positive clones. Sequencing of the positive clones indicated successful insertion of the BcPAD gene into the correct position of the pET28a vector, demonstrating that the *E. coli* BL21(DE3)-pET28a-*BcPAD* recombinant strain was constructed successfully. ProtParam analysis indicated that the theoretical isoelectric point (pI) of BcPAD is 4.93, with an associated molecular weight of 19.63 kDa. The instability index (II) was calculated to be 27.83, and the grand average of hydropathicity (GRAVY) was −0.564, indicating that the protein is both hydrophilic and stable.

An amino acid sequence alignment of BcPAD with other reported phenolic acid decarboxylases revealed that BcPAD shares the highest sequence identity of 79.50% with BsPAD from *Bacillus subtilis* (PDB ID: 4ALB) [37]. Additionally, BcPAD displays sequence identities of 77.78% with BaPAD from *Bacillus atrophaeus* (GenBank accession number: AKL86192.1) [38], 77.50% with BpPAD from *Bacillus pumilus* (PDB ID: 3NAD) [39], 72.56% with LpPDC from *Lactobacillus plantarum* (PDB ID: 2W2A) [34], and 69.94% with LvPAD from *Lactobacillus vermolensis* (GenBank accession number: WIW58156.1) [2]. Notably, the twelve key active sites of phenolic acid decarboxylases, as previously reported [21,40,41], are highly conserved among these enzymes (Figure 5C). These results suggest that BcPAD is a member of the phenolic acid decarboxylase family and may exhibit similar catalytic functions.

### 3.5. Expression and Purification of Recombinant BcPAD

**Figure 6:**
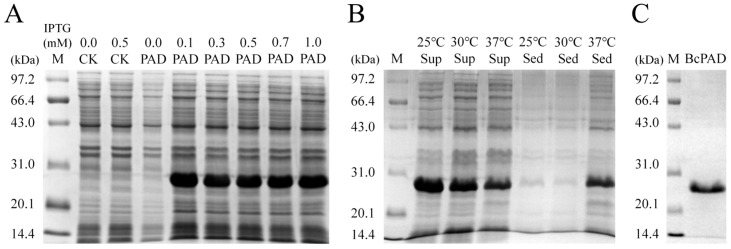
SDS-PAGE analysis of recombinant BcPAD. (**A**) Induction of recombinant BcPAD with different concentrations of IPTG. CK: *E. coli* BL21(DE3)-pET28a. (**B**) Induction of recombinant BcPAD at different temperatures. Sup: supernatant; Sed: precipitate. (**C**) Purification of recombinant BcPAD. M: protein molecular weight marker.

SDS-PAGE analysis indicated that the molecular weight of recombinant BcPAD is 23.08 kDa (Figure 6), which is slightly higher than the 19.2 kDa reported for BsPAD-Q58 by Zhang et al. [42]. This increase can be attributed to the fusion of a 33-amino acid histidine tag at the N-terminus of recombinant BcPAD, contributing an additional 3.45 kDa to its theoretical molecular weight. There was no significant difference in the expression levels of BcPAD induced by different IPTG concentrations (Figure 6A), and a large amount of target protein was produced after 3 h of induction at a lower IPTG concentration (final concentration of 0.1 mmol/L). Protein distribution analysis revealed that BcPAD mainly exists as a soluble protein at different induction temperatures (Figure 6B), and it was present in both the supernatant and precipitate when induced at 37 °C. Previous studies have confirmed that low-temperature induction can reduce the rate of protein expression, facilitating proper protein folding and thereby increasing the yield of soluble proteins [43,44]. Consequently, induction at lower temperatures is more advantageous for the expression of soluble BcPAD. The soluble protein in the supernatant was purified using Ni-NTA affinity chromatography to obtain purified BcPAD (Figure 6C), with a protein concentration of 0.1354 mg/mL.

### 3.6. Effects of pH and Temperature on the Activity and Stability of BcPAD

**Figure 7:**
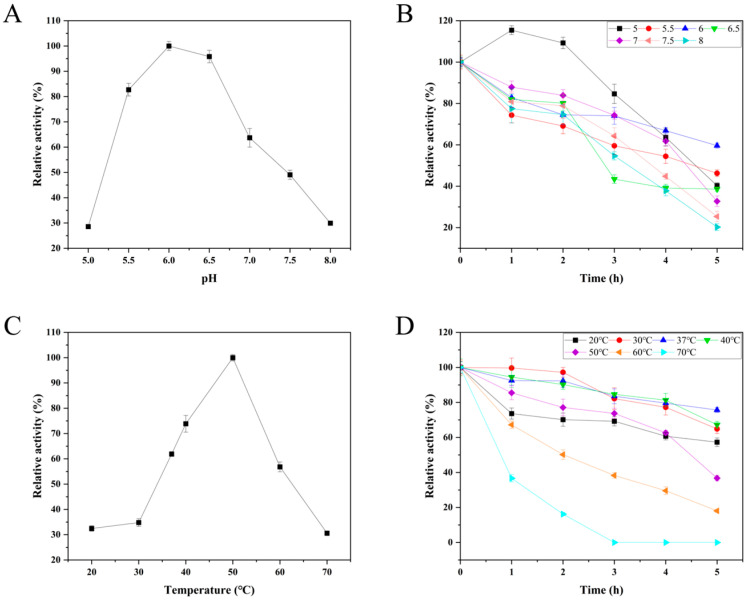
Enzymatic characteristics of BcPAD under different temperature and pH conditions. (**A**) Optimal pH of BcPAD. (**B**) pH stability of BcPAD. (**C**) Optimal temperature for BcPAD. (**D**) Temperature stability of BcPAD.

BcPAD exhibits activity across a pH range of 5.0 to 8.0, with an optimal pH of 6.0 (Figure 7A), which is consistent with the optimal pH of LbPAD from *Lactobacillus brevis* [10] and is close to the 5.7 of AlPAD from *Aspergillus luchuensis* [45]. Although the activity of BcPAD significantly decreases under both acidic and alkaline conditions, it retains relative activities of 28.58% and 29.94% at pH 5.0 and pH 8.0, respectively, which differs from the result that LbPAD loses activity under pH 8.0 conditions [10]. After treatment for 5 h within the pH range of 5.0 to 6.0, the relative activity of BcPAD remains above 40% (Figure 7B). Under pH 8.0 conditions, enzyme activity is only about 20% after 5 h. These results suggest that a mildly acidic environment may be beneficial for the catalytic activity of BcPAD.

It has been reported that the structure and kinetics of enzymes may change with variations in temperature, thereby affecting their catalytic efficiency [46]. As shown in Figure 7C, BcPAD exhibits activity across a temperature range of 20–70 °C, with an optimal reaction temperature of 50 °C, which is consistent with the findings reported by Ni et al. [47] and is higher than the optimal temperatures of 35 °C for LvPAD from *L*. *vermolensis* [2] and 37 °C for BlPAD from *B*. *licheniformis* [36]. In the temperature range of 30–40 °C, the relative enzyme activity gradually decreases with extended incubation time, but remains above 60% after 5 h. At the optimal temperature of 50 °C, the relative enzyme activity maintained 63% even after 4 h. When the temperature is raised to 70 °C, the relative activity remains at 16% for 2 h but is completely inactivated after 3 h. Overall, BcPAD exhibits a certain degree of thermal tolerance.

### 3.7. Effects of Metal Ions and Chemical Reagents on the Activity of BcPAD

**Figure 8:**
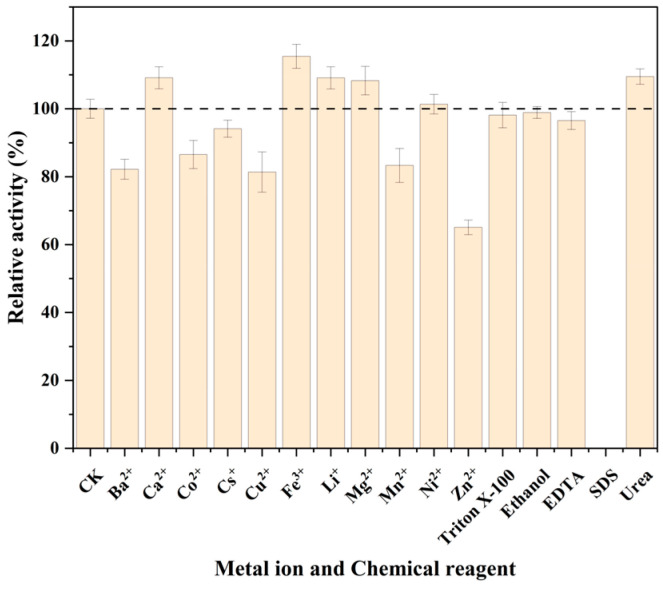
Effects of metal ions and chemical reagents on BcPAD activity.

The effects of metal ions and chemical reagents on BcPAD activity were evaluated using FA as the substrate (Figure 8). Ba^2+^, Co^2+^, Cu^2+^, Mn^2+^, and Zn^2+^ exhibited varying degrees of inhibition, with Zn^2+^ exerting the most pronounced inhibitory effect, reducing the relative activity of BcPAD by 34.92%. In contrast, Ca^2+^, Fe^3+^, Li^+^, and Mg^2+^ enhanced BcPAD activity, with Fe^3+^ being the most effective, increasing the relative activity to 115.36%. These metal ions were predominantly inhibitory in studies on LvPAD by Zhao et al. [2] and on BsPAD-Q58 by Zhang et al. [42], respectively, which may be attributable to the use of PCA as the substrate and differences in the sources of the phenolic acid decarboxylases. Triton X-100, ethanol, and EDTA exhibited no significant effect on BcPAD activity. However, SDS completely inactivated BcPAD, consistent with the findings for BaPAD reported by Li et al. [38]. Urea exhibited a moderate activating effect, increasing the relative activity to 109.49%, similar to the findings for LbPAD reported by Landete et al. [10].

### 3.8. Substrate Specificity and Kinetic Parameters of BcPAD

BcPAD exhibited significant substrate specificity and catalytic efficiency differences among various phenolic acid substrates. Substrate specificity analysis showed that BcPAD had the highest specific activity for PCA (460.6 ± 18.3 U/mg), identifying PCA as its preferred substrate (Table 3). The specific activity for CA was 145.9 ± 2.9 U/mg, lower than that for PCA, whereas that for FA was only 34.5 ± 1.1 U/mg. No enzymatic activity was detected for SA, suggesting that BcPAD scarcely catalyzes this substrate, consistent with a previous report [10]. These results indicate that BcPAD has significant selectivity for structurally similar phenolic acids and prefers to catalyze the conversion of PCA.

The kinetic parameters further supported this substrate preference (Table 4). PCA exhibited the highest *Vmax* and *Kcat* values (11.3 ± 0.2 mM/min and 322.1 ± 4.1 s^−1^, respectively) and the highest *Kcat*/*Km* (12.1 ± 1.4 mM^−1^·s^−1^), demonstrating that BcPAD achieves its highest overall catalytic efficiency toward PCA under the optimal reaction conditions. In contrast, although FA had the lowest *Km* value (4.7 ± 0.7 mM), reflecting the highest affinity for the enzyme, its *Vmax* and *Kcat* were significantly lower (0.5 ± 0.0 mM/min and 13.6 ± 0.2 s^−1^), resulting in a *Kcat*/*Km* value of only 2.9 ± 0.4 mM^−1^·s^−1^ and, consequently, lower overall catalytic efficiency. CA exhibited *Km*, *Vmax*, *Kcat*, and *Kcat*/*Km* values that intermediate between those of PCA and FA. In summary, the combined substrate specificity and kinetic analyses demonstrated that BcPAD has a clear catalytic advantage for PCA, while its catalytic performance for FA and CA was comparatively weaker, similar to previous reports [48,49].

### 3.9. Structural Analysis of BcPAD

**Figure 9:**
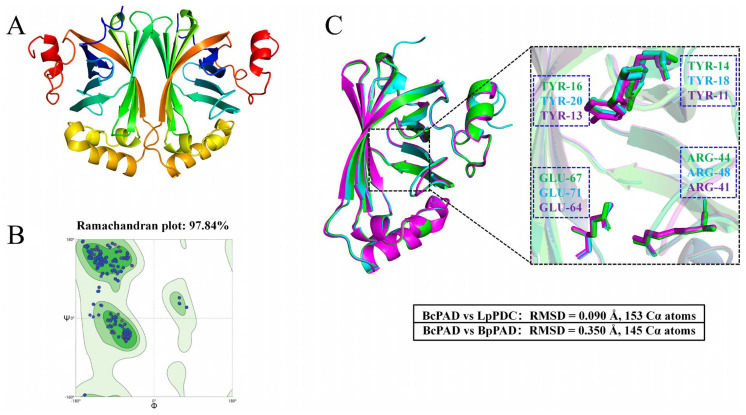
Three-dimensional structure and ramachandran plot assessment of BcPAD. (**A**) Three-dimensional structure of BcPAD. (**B**) Results of Ramachandran plot assessment. (**C**) Superposition of monomer structures; green represents BcPAD, blue represents LpPDC (PDB ID: 2W2A), and purple represents BpPAD (PDB ID: 3NAD).

Homology modeling based on crystal structures is an effective approach for predicting protein tertiary structures, which is helpful for analyzing the molecular mechanisms of enzymatic property differences at the structural level [50]. The above results indicate that BcPAD exhibits significant differences in its ability to catalyze different phenolic acid substrates. To explore the possible structural basis of these differences, homology modeling and molecular docking analyses were carried out. The results showed that BcPAD forms a homodimer (Figure 9A), consistent with the dimeric structures previously reported for related PAD-family enzymes [40,51,52]. Ramachandran plot analysis revealed that over 97% of the residues of BcPAD are positioned within favored regions (Figure 9B), supporting the overall reliability of the model. Furthermore, the three-dimensional structure of BcPAD is highly similar to those of LpPDC (PDB ID: 2W2A) and BpPAD (PDB ID: 3NAD), with Cα root-mean-square deviation (RMSD) values of 0.090 Å over 153 aligned Cα atoms and 0.350 Å over 145 aligned Cα atoms, respectively (Figure 9C). Only minor differences were observed at the N-terminus, C-terminus, and in several loop regions.

A more detailed comparison of the active-site region further supported the mechanistic relevance of the BcPAD model. In the experimentally characterized LpPDC structure (PDB ID: 2W2A) [40], TYR-18, TYR-20, ARG-48, and GLU-71 were identified as key functional residues, and structural and mutational analyses suggested that GLU-71 participates in proton transfer, whereas TYR-18 and TYR-20 contribute to substrate orientation and CO_2_ release during decarboxylation. In the closely related BpPAD structure (PDB ID: 3NAD) [39], the corresponding residues TYR-11, TYR-13, ARG-41, and GLU-64 were structurally conserved, further indicating preservation of the catalytic framework among phenolic acid decarboxylases. In the BcPAD model, the corresponding conserved TYR-14, TYR-16, ARG-44, and GLU-67 residues were positioned in the same narrow active-site cleft (Figure 9C), suggesting that BcPAD retains the catalytic architecture required for substrate positioning and decarboxylation. These conserved active-site features provided a useful structural context for interpreting the docking results of BcPAD.

**Figure 10:**
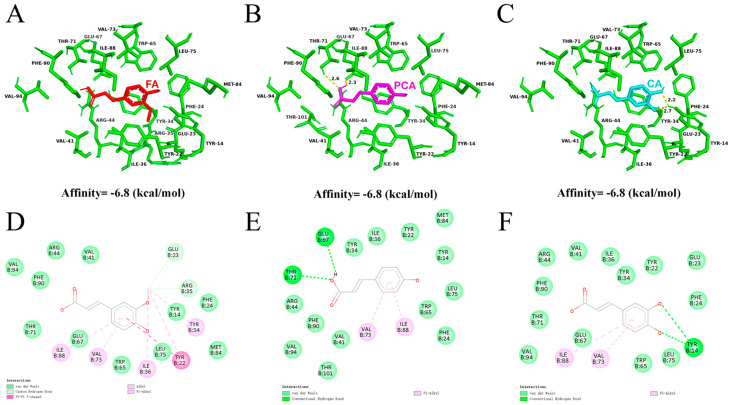
Molecular docking analysis of BcPAD with different phenolic acids. (**A**) Three-dimensional structure of BcPAD interacting with FA. (**B**) Three-dimensional structure of BcPAD interacting with PCA. (**C**) Three-dimensional structure of BcPAD interacting with CA. (**D**) Two-dimensional structure of BcPAD interacting with FA. (**E**) Two-dimensional structure of BcPAD interacting with PCA. (**F**) Two-dimensional structure of BcPAD interacting with CA.

To further investigate the possible structural basis for the differences in the catalytic performance of BcPAD toward various phenolic acid substrates, molecular docking analyses were performed to examine the predicted binding modes of PCA, CA, and FA to BcPAD (Figure 10). The results showed that all three substrates exhibited the same minimum binding energy (−6.8 kcal/mol), suggesting comparable predicted binding strengths at the docking level, although their binding orientations and interaction patterns differed substantially. Therefore, the following interpretations should be regarded as hypotheses based primarily on the predicted binding orientations and interaction patterns rather than as firm mechanistic conclusions. Among the three substrates, FA interacted with the largest number of residues, involving 18 amino acid residues (Figure 10A), including 11 van der Waals interactions, 2 carbon–hydrogen bonds, and 5 hydrophobic interactions, such as Pi–Pi T–shaped, Alkyl, and Pi–Alkyl (Figure 10D). These observations suggest that FA binds tightly within the active site primarily through nondirectional hydrophobic interactions and van der Waals forces but lacks specific hydrogen bonds with key active-site residues. Previous studies have shown that binding stability can be enhanced by hydrophobic interactions, hydrogen bonding, and electrostatic interactions [53,54]. The “multipoint hydrophobic restraint” mode of FA may therefore enhance binding stability while simultaneously limiting substrate conformational adjustment during catalysis, which is consistent with its kinetic profile of high affinity but low turnover and agrees with the findings of Zhou et al. [12].

By contrast, PCA interacted with 17 amino acid residues (Figure 10B), including 13 involved in van der Waals interactions, and formed two key hydrogen bonds with GLU-67 and THR-71, together with Pi–Alkyl hydrophobic interactions with VAL-73 and ILE-88 (Figure 10E). Previous studies have indicated that the hydrogen-bonding network formed by the substrate within the active site plays a crucial role in regioselectivity [55]. Although its docking energy was identical to those of the other two substrates, this interaction pattern may favor a binding orientation in which PCA is more suitably aligned within the active site. Such an orientation could potentially facilitate catalysis, but this remains hypothetical because docking does not directly evaluate productive binding or transition-state stabilization. Nevertheless, this interpretation is consistent with the high *Vmax*, *Kcat*, and *Kcat*/*Km* values observed for PCA. CA displayed a binding pattern intermediate between those observed for FA and PCA. It interacted with 16 amino acid residues (Figure 10C), including 13 van der Waals interactions, and formed similar Pi–Alkyl hydrophobic interactions with VAL-73 and ILE-88; however, its hydrogen bonds were formed with TYR-14 rather than with GLU-67 and THR-71 (Figure 10F). This result may suggest that although CA can achieve relatively stable binding in the active site, the spatial orientation of its key functional groups may be less optimally aligned with the catalytic residues than that of PCA, resulting in intermediate transition-state stability and catalytic efficiency.

These results suggest that substrate-dependent differences in BcPAD activity may be associated with variations in the hydrogen-bonding network, hydrophobic interactions, and the orientation and conformational compatibility of the substrates within the active site. These observations are generally consistent with previous structural studies [12,56]. However, these interpretations should be considered tentative pending further experimental validation.

## 4. Conclusions

In this study, *B. coagulans* JN11 was identified as the highest 4-EG-producing strain among the tested isolates, with a yield of 271.6 ± 2.7 μg/L. Its growth characteristics suggested adaptation to mildly acidic conditions and moderate temperatures. The phenolic acid decarboxylase gene, *BcPAD*, was successfully cloned from *B*. *coagulans* JN11 and heterologously expressed in *E. coli* BL21(DE3). Recombinant BcPAD showed optimal activity at pH 6.0 and 50 °C, retained considerable stability under mildly acidic conditions and at moderate temperatures, and responded differentially to metal ions and chemical reagents. Ca^2+^, Fe^3+^, Li^+^, Mg^2+^, and urea enhanced enzyme activity, whereas Zn^2+^ markedly inhibited enzyme activity and SDS completely inactivated the enzyme. Substrate specificity and kinetic analyses demonstrated that BcPAD preferentially catalyzed PCA, whereas CA and FA were converted less efficiently, and no activity was detected toward SA. Notably, although FA exhibited a lower *Km*, its much lower turnover indicated that catalytic efficiency was not determined solely by substrate affinity. Structural modeling and molecular docking suggested that these substrate-dependent differences may be associated with variations in substrate orientation and hydrogen-bonding interactions within the active site. Overall, these findings improve our understanding of substrate recognition and catalysis by BcPAD from *B. coagulans*. Although 4-EG production was observed during fermentation by strain JN11, BcPAD was biochemically characterized as a phenolic acid decarboxylase likely involved in the upstream formation of vinyl derivatives, whereas the final formation of 4-EG in strain JN11 likely requires an additional downstream reduction step. The present results provide a basis for further investigation of the contributions of strain JN11 and BcPAD to volatile phenol formation during Baijiu production.

## Figures and Tables

**Table 1:** Strains and plasmids used in this study.

Strains/Plasmids	Description	Source
JXQ2	The strain was isolated from the high-temperature *Daqu*	Lab stock
JXQ5	The strain was isolated from the high-temperature *Daqu*	Lab stock
NXQ4	The strain was isolated from the medium-temperature *Daqu*	Lab stock
JN2	The strain was isolated from the mud of the liquor cellar	Lab stock
JN11	The strain was isolated from the mud of the liquor cellar	Lab stock
*E. coli* BL21(DE3)	Host for gene expression	TransGen
*E. coli* BL21(DE3)/pET-28a	Expression strain carrying plasmid pET-28a	This work
*E. coli* BL21(DE3)/pET-28a-*BcPAD*	Expression strain carrying plasmid pET-28a-*BcPAD*	This work
pET-28a(+)	*E. coli* expression vector, Kan^r^	TaKaRa (Dalian, China)
pET-28a-*BcPAD*	pET-28a(+) derivative harboring *BcPAD* gene, Kan^r^	This work

**Table 2:** Physiochemical characteristics of the JN11 strain.

Biochemical Test	Test Result	Biochemical Test	Test Result
Glucose fermentation	+	Gelatin hydrolysis	+
Sucrose fermentation	+	Starch hydrolysis	+
Lactose fermentation	+	Nitrate reduction	+
Mannitol fermentation	−	Citrate utilization	−
Catalase activity	+	VP test	+
Cellulase activity	+	Indole test	−
Proteinase activity	+	Anaerobic growth	+

+ indicates positive, − indicates negative.

**Table 3:** Substrate specificity of BcPAD.

Substrates	Specific Activity (U/mg)	Relative Activity (%)
Ferulic acid	34.5 ± 1.1	7.48
*p*-Coumaric acid	460.6 ± 18.3	100
Caffeic acid	145.9 ± 2.9	31.68
Sinapic acid	ND	ND

Different phenolic acids at a concentration of 80 mM were used as substrates for the assay, and all other assay conditions were identical. ND indicates that no activity was detected.

**Table 4:** Kinetic parameters of BcPAD.

Kinetic Parameters	Substrates		
	Ferulic Acid	*p*-Coumaric Acid	Caffeic Acid
*Km* (mM)	4.7 ± 0.7	26.7 ± 3.2	10.2 ± 1.4
*Vmax* (mM/min)	0.5 ± 0.0	11.3 ± 0.2	2.1 ± 0.1
*Kcat* (s^−1^)	13.6 ± 0.2	322.1 ± 4.1	60.0 ± 1.3
*Kcat*/*Km* (mM^−1^·s^−1^)	2.9 ± 0.4	12.1 ± 1.4	5.9 ± 0.8

Kinetic parameters were determined at pH 6.0 and 50 °C with substrate concentrations ranging from 5 to 160 mM. *Vmax* values were converted from U/mg to mM/min using the final enzyme concentration in the reaction mixture (0.01354 mg/mL). *Kcat* values were calculated based on a monomer molecular weight of 23.08 kDa.

## Data Availability

The original contributions presented in the study are included in the article/Supplementary Materials; further inquiries can be directed to the corresponding author.

## Supplementary Materials

The following supporting information can be downloaded at: https://www.mdpi.com/article/10.3390/microorganisms14061338/s1, Table S1: GC-MS products of the JN11 strain after 7 days of fermentation.
