# Supplementary figures and images for: Screening of a 4-Ethylguaiacol-Producing Bacillus coagulans JN11 and Biochemical Characterization of Its Phenolic Acid Decarboxylase BcPAD
Source: Microorganisms. 2026 Jun 15;14(6):1338. doi: 10.3390/microorganisms14061338 (PMC13304128; doi:10.3390/microorganisms14061338)

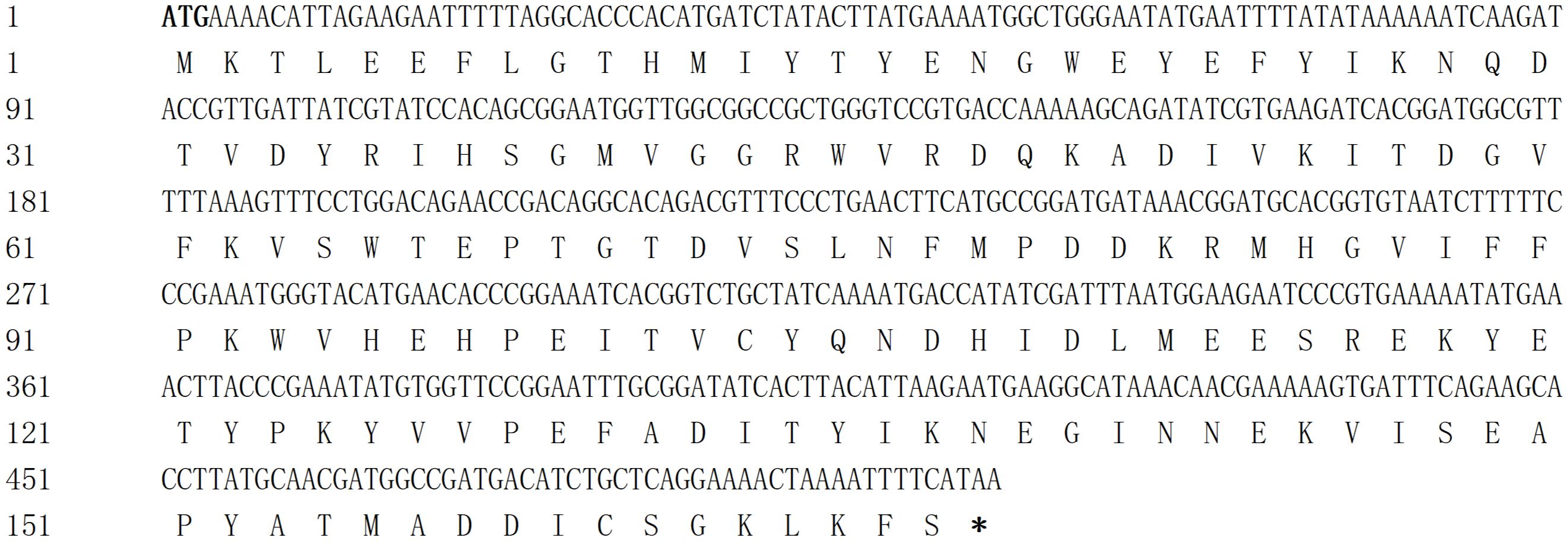

Supplement: Supplementary file 1 [file microorganisms-14-01338-s001.zip › Figure S1.png]
